# Accumulation and ecotoxicological risk assessment of heavy metals in surface sediments of the Olt River, Romania

**DOI:** 10.1038/s41598-022-04865-0

**Published:** 2022-01-18

**Authors:** Andreea Maria Iordache, Constantin Nechita, Ramona Zgavarogea, Cezara Voica, Mihai Varlam, Roxana Elena Ionete

**Affiliations:** 1 National Research and Development Institute for Cryogenics and Isotopic Technologies—ICSI Rm. Valcea, 4 Uzinei Street, 240050 Rm. Valcea, Valcea, Romania; 2 National Institute for Research and Development in Forestry “Marin Drăcea” Calea Bucovinei, 73 bis, 725100 Câmpulung Moldovenesc, Romania; 3 National Institute for Research and Development of Isotopic and Molecular Technologies, 67-103 Donat St, 400293 Cluj-Napoca, Romania

**Keywords:** Natural hazards, Chemistry, Environmental chemistry, Environmental monitoring

## Abstract

Heavy metal pollution of river freshwater environments currently raises significant concerns due to the toxic effects and the fact that heavy metal behavior is not fully understood. This study assessed the contamination level of eight heavy metals and trace elements (Cr, Ni, Cu, Zn, As, Pb, Cd, and Hg) in the surface sediments of 19 sites in 2018 during four periods (March, May, June, and October) in Olt River sediments. Multivariate statistical techniques were used, namely, one-way ANOVA, person product-moment correlation analysis, principal component analysis, hierarchical cluster analysis, and sediment quality indicators such as the contamination factor and pollution load index. The results demonstrated higher contents of Ni, Cu, Zn, As, Pb, Cd, and Hg, with values that were over 2.46, 4.40, 1.15, 8.28, 1.10, 1.53, and 3.71 times more, respectively, compared with the national quality standards for sediments. We observed a positive significant statistical correlation (*p* < 0.001) in March between elevation and Pb, Ni, Cu, Cr, and Zn and a negative correlation between Pb and elevation (*p* = 0.08). Intermetal associations were observed only in March, indicating a relationship with river discharge from spring. The PCA sustained mainly anthropogenic sources of heavy metals, which were also identified through correlation and cluster analyses. We noted significant differences between the Cr and Pb population means and variances (*p* < 0.001) for the data measured in March, May, June, and October. The contamination factor indicated that the pollution level of heavy metals was high and significant for As at 15 of the 19 sites. The pollution load index showed that over 89% of the sites were polluted by metals to various degrees during the four periods investigated. Our results improve the knowledge of anthropogenic versus natural origins of heavy metals in river surface sediments, which is extremely important in assessing environmental and human health risks and beneficial for decision-maker outcomes for national freshwater management plans.

## Introduction

Heavy metal (HM) contents and fluctuations threaten aquatic environments due to their availability, toxicity and persistence^1^. Anthropic sources represent the primary pathway for HM occurrence in water and sediments^2^. Human activities repeatedly increase contamination in aquatic ecosystems, and various amounts of metals are assimilated and deposited in water, sediment, and biota^3^. The accumulation of HMs in sediments by complex physicochemical adsorption mechanisms depends on the sediment matrix and the adsorption properties of the compounds^4^. Furthermore, the fluctuation of pollutants in the water and sediments is conditioned by multiple environmental conditions, such as discharges, temperature, pH, and organic matter^5^. HMs discharged into the environment can be deposited in sediments, allowing the prediction of future environmental change based on past and present monitoring^6^.

Nonessential and some toxic metals (e.g., Hg, Cd, As, and Pb) have been subjected to intense study due to their effects on living organisms^7^. On a global scale, arsenic (As) is the highest contaminant found in drinking water^8^. Several hypotheses have been formulated to explain the changing behavior of As in water induced by environmental factors. The predominant As species are As(V) and As(III), and their presence in an aqueous environment varies with pH and redox conditions^9^. Fe-oxide minerals in the middle‒acidic pH range induce strong arsenate adsorption, and desorption has been observed at pH ~ 8^10^; these processes can be associated with As mobility^11^. HM accumulation has been less studied in the context of increasing climate effect changes, anthropogenic activities and modifying nutrient behavior^12,13^. Even so, only an insignificant proportion of loaded metal ions remain dissolved in water; the rest are transported to and accumulate in sediments^14^. Compared with water analysis, sediment chemical profiling can provide a more profound overview of the potential sources and sinks for chemical compounds mainly associated with the grain sediment fraction (clay and silt particles) and particulate organic carbon^15^. Sediment deposits are not precisely defined. They are considered complex associations of gases, dissolved compounds, and organic materials, resulting from various sources controlled by physicochemical and biological processes and factors^16^. Suspended particles and sediments transported along rivers create deposits in riverine lakes that accumulate pollution over time, and these patterns are reliable for assessing time-integrated trends^17^.

Extensive investigations of HM pollution in rivers have been conducted worldwide^18^. In recent decades, increases in industrial development, urbanization, and human activity have led to large amounts of wastewaters and solid wastes containing HMs being discharged in Romanian rivers^19^. Several studies have presented the HM enrichment of water and sediments for those rivers^20–22^. Most of them have focused on the Olt River since it has been subjected to numerous contamination sources and is the most important tributary of the Danube River^19,23,24^. The Olt River receives waste, mining, industry, and other anthropic activity inputs^19^. Iordache et al. demonstrated high contamination with As and moderate sediment pollution with Ni^25^. Samples collected from nine sites in February 2015 indicated heavy pollution according to the pollution load index in eight of those nine areas. Even the Nemerow Pollution Index showed the same level of sediment contamination of HMs. Past studies have attributed severe ecological risk to sites located downstream by the municipality of Râmnicu Vâlcea, which is associated with contamination sources from wastewaters and industrial discharges from the Oltchim chlor-alkali plant^23,26^. However, few studies have focused on monitoring and assessing the pollution level of surface sediments in the Olt River basin.

This study aimed (i) to assess the current contamination status of Zn, Cr, Cu, Ni, Pb, As, Cd, and Hg and their temporal-spatial distribution in the surface sediment of the middle and lower reaches of the Olt River; (ii) to identify potential sources of surface sediment contamination with HMs; (iii) to quantify the interaction of the HMs in the sediments and the elevation of the site; and finally (iv) to evaluate the ecological risk using Geoaccumulation Index (*I*_*geo*_), Nemerow Pollution Index (*PI*), Potential Ecological Risk (*RI*) and pollution load index (*PLI*) in the Olt River ecosystem. The present study focused on investigating HM profiles in the surface sediments of 19 sites in 2018 during four periods (March, May, June, and October), representing the first systematic study in the area. Our results indicate managers should pay close attention to the coming years since the interannual variability highlighted vast amounts of some metals occurring currently compared with the past decade, even if several mitigation actions are in progress. The outcom es of this study will serve as a reference for Romania, enhance understanding internationally, and help inform governmental strategies for addressing environmental pollution.

## Material and methods

The surface sediment samples were collected from 19 sites located in the middle and lower reaches of the Olt River during four field trips in March (T1), May (T2), June (T3), and October (T4) 2018 (Supplementary Table 1). Eight significant HMs and trace elements (Zn, Cr, Cu, Ni, Pb, As, Cd, and Hg) were analyzed. The data are presented as the mean (*µ*), range of elements, standard deviation (*sd*), coefficient of variation (*cv*), and interquartile range (*q3*‒*q1*) to estimate the trend and pattern of variation among sampling sites and periods. One-way ANOVA and post hoc Bonferroni tests were used to assess differences in seasonal concentrations of metals in sediments. In addition, the Levene test for equal variance was evaluated. Finally, multivariate analyses, including correlation analysis (CA), principal component analysis (PCA), hierarchical cluster analysis (HCA), and linear regression (LR), were used to determine possible intercorrelation between elements and the element group. PCA was used to assess contamination sources predicted by correlation analysis using varimax rotation with Kaiser normalization. Factor loadings for HMs in sediments were extracted based on engine values higher than 1. HCA was performed to find similarities among metals using Euclidian distance. The statistical analyses were carried out using SPSS 27 (IBM SPSS Statistics, IBM Corp., USA) and R v4.1.0^27^.

## Results and discussions

### Sediment quality

Overall, the average concentration of the HMs had a decreasing order of As > Zn > Pb > Ni > Cr > Cu > Cd > Hg. HMs become more toxic to the Earth spheres (biosphere, atmosphere, hydrosphere, and lithosphere), and the effects are more evident, especially on human health^28^. Only a limited number of governments have shown concern for minimizing HMs in ecosystems^29^. The World Health Organization (Joint FAO/WHO Expert Committee on Food Additives, JECFA)^30^ has recommended strict limits for exposure to, or the lifetime of, consumer products that contain a certain degree of contamination. Several HMs are monitored and banned (Hg, Pb, Cd, As), as their level of toxicity is very high^31,32^. In comparison, other elements (e.g., Cr and Zn) do not have very strict regulations^30^. Recent studies demonstrate that constant exposure, even at lower levels, increases the risk of respiratory tract cancer^33^ and cardiovascular or neurodegenerative diseases^34,35^.

**Figure 1:**
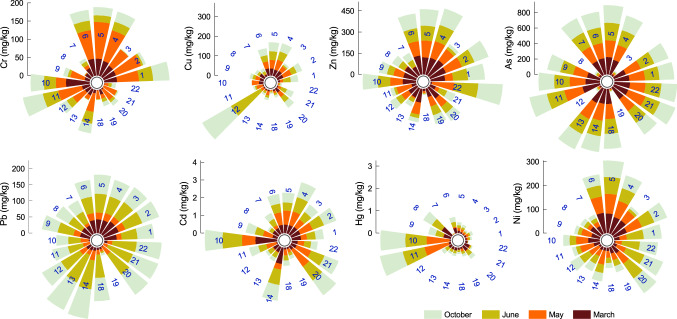
Spatial–temporal distribution of heavy metals contamination level in the 2018 Olt River sediments.

Basic statistics for the investigated elements (Cr, Ni, Cu, Zn, As, Pb, Cd, and Hg) in sediments across the middle and lower Olt River showed the following mean concentration levels (*µ*): 25.19, 42.20, 24.13, 73.78, 163.97, 39.44, 0.49, and 0.19 mg/kg, respectively (Fig. 1). The HM concentration ranged from 0.09 to 100.66 mg/kg for Cr, 5.68‒86.31 mg/kg for Ni, 0.12‒176.00 mg/kg for Cu, 3.4‒172.90 mg/kg for Zn, 10‒240.14 mg/kg for As, 0.50‒94.20 mg/kg for Pb, 0.01‒1.23 mg/kg for Cd, and 0.00‒1.11 mg/kg for Hg. Compared to the national thresholds^36^, most of the site values were unacceptably high, exceeding their background values by factors of 2.46 (Ni), 4.40 (Cu), 1.15 (Zn), 8.28 (As), 1.10 (Pb), 1.53 (Cd), and 3.71 (Hg). These results indicate that the origins of the metals investigated are caused more by anthropogenic sources than by orogenic sources^37^. We compared our results to the worldwide published HM levels in surface sediments and observed that the concentrations of Cr, Ni, Cu, Zn, Pb, Cd, and Hg measured in the Olt River in 2018 were comparable to those mentioned in Supplementary Table 2. Only the As concentration in the Olt River sediments had a very high contamination level related to the Pra Basin^35^, the Lijiang River^39^, Lake Taihu^40,41^, the Seine River^42,43^, the Odra River^44^, the Danube River^45^, the Hackensack and East Riser Rivers^46^, the Kuril-Kamchatka^47^, and the Lower Indus^48^. Even so, the As content in Olt River sediments was much lower than that of semiarid climate mining areas, e.g., Villa de la Paz-Matehuala, Mexico (17 384 mg/kg)^49^, and Cabezo Rajao, Spain (314.7 mg/kg)^50^. Mining activities appear to be the highest source of As pollution, and As enters river ecosystems by wind, flash floods in the hot season, from water discharges in spring after snowmelt, or from accidental releases^51^.

However, the mean concentration of As reported in two studies from the Pra Basin of Ghana, a region with gold mines, showed reduced contamination^38,52^. Both studies showed comparable results in two different sampling periods (0.15 mg/kg and 0.714 mg/kg), indicating no exceptional events causing point source pollution and explaining comparable values in time (low variability). However, in the Olt River case, the difference in the concentration level for samples collected in 2018 and 2019 was significant within the same sampling areas, ranging from a maximum value of 240.14 mg/k in the first year to 8.35 mg/kg in the second year^19^. We noted the highest concentration of As (mg/kg) at site #11 (T1 = 232, T2 = 240.14, T3 = 228.7, T4 = 231.4); for the same location in 2019, the values ranged between 0.238 and 7.79 mg/kg. It is worth mentioning that the As maximum value did not exceed 29.4 mg/kg at sites #3, #8, #12, or #19; at the other sites, the concentration level was high, comparable with that at site #11. Extreme amounts of HMs are deposited on the bottom river layers and remobilized under different environmental conditions^53^.

### Spatial and temporal distribution of HMs in sediments

We noted that the HM concentration fluctuated from upstream sites (#1) until the Olt River overflowed into the Danube (#12). The spatial variation in contaminant levels for each sample site and period is illustrated in Fig. 1. The coefficients of variation (*cv*) for Cr, Ni, Cu, Zn, As, Pb, Cd, and Hg had respective ranges of 66‒90%, 41‒66%, 70‒135%, 44‒63%, 47‒48%, 35‒79%, 48‒65%, and 81‒208%, indicating that these elements had uneven spatial and temporal distributions. A high HM concentration range, corroborated with a high coefficient of variation amplitude, showed substantial anthropogenic influence^54^. Statistically, the significance of a low coefficient of variation in As, for example, suggests a relatively stable variation in HM contamination levels in sediment samples and not necessarily a natural background. The analyzed dataset showed significant differences between the Olt River sampling sites from the middle reaches (#1‒#10) and the lower reaches (#11‒#22) only for Cr and Zn (*p* < 0.01). This result indicates there are more HMs in the middle reaches than at sites in the lower reaches. The higher level of HM contamination can be explained through various input sources of anthropically induced pollution, such as dams from power plants that create specific environments by restricting the river flow velocity^13^. River hydrodynamics regulate desorption and absorption reactions and can also be responsible for high contaminant level variability^55^. Other authors have attributed the higher concentration of some metals (e.g., Hg and Pb) to the Râmnicu Vâlcea chemical chlor-alkali plant^23^. This rationale does not explain similar or high amounts of pollutants from the sites located far upstream, starting with site #1 (Fig. 1). The higher values measured in most investigated locations can result after spring floods and extreme events that transport and deposit HMs released from industrial and municipal waste, mining, and agricultural practices^13^.

Chromium is a mutagenic, highly toxic, and carcinogenic element and it has been extensively studied because of its capacity to migrate and transform in surface sediments after long-term contamination. Chromium naturally occurs in two oxidation states: Cr(VI), including the highly soluble oxyanion, and Cr(III), which is less toxic and less mobile. Cr(VI) is involved in redox reactions, absorption–desorption, and oxidation forms, and it control Cr toxicity and mobility^56,57^. In our case, the range of maximum concentrations for chromium measured in each period oscillated between 42.0 mg/kg (T3) and 98.9 mg/kg (T2), and extreme values were achieved at sites #10 (T1), #5 (T2) and #11 (T3, T4). In addition, we observed significant differences between the Cr population means (*F* = 7.61, *p* < 0.0001) and population variances (*F* = 4.97, *p* < 0.003) of the four periods investigated. Chromium originates from untreated industrial emissions and domestic wastewater discharge released into the Olt River. The mean HM statistics for each sampling period showed higher values in T1 (22.94 mg/kg) and T2 (43.57 mg/kg) than in T3 (15.14 mg/kg) and T4 (17.84 mg/kg).

Even for Pb, significant differences between populational means (*F* = 29.93, *p* < 0.0001) and populational variance (*F* = 4.94, *p* = 0.003) were calculated. The Pb contaminant level varied from 28.1 mg/kg (T2) to 86.6 mg/kg (T3, T4), with maximum values at sites #3 (T1), #14 (T2), and #13 (T3, T4). The sampling periods can be associated with spring (March, May), summer (June), and autumn (October). Each period is characterized by specific climatic conditions, including temperature, precipitation, and evapotranspiration, which can induce a specific regime of organic matter, pH, and the association between metals^58^. The population means and variance of the four periods investigated did not vary significantly in the cases of Ni, Cu, Zn, As, Cd, or Hg, for which the contaminant level (mg/kg) ranges were 66.93‒80.63, 36.09‒175.80, 124.89‒165, 217.66‒226.74, 0.90‒1.17, and 0.51‒1.09, respectively. One-way ANOVA statistical analysis suggested no relationship between the spatial location and Ni, Cu, As, Pb, Cd, and Hg.

The nickel concentration in the Olt River sediments had relatively comparable mean values (in mg/kg with standard deviation) in all four periods investigated (T1 = 38.54 ± 25.72, T2 = 45.57 ± 18.84, T3 = 42.26 ± 24.09, T4 = 42.46 ± 24.42). The Ni concentration was higher than the national safety guideline of 35 mg/kg at 45 randomly distributed sites along with spatial position and sampling periods, indicating natural and human-induced sources. The natural presence of Ni in the river environment can be associated with watering soils and rocks and atmospheric deposition. A strong correlation was presented with acid-volatile sulfide (AVS), total organic carbon (TOC), Fe, and Mn exchange capacity (CEC)^59^. The Ni geochemistry is not fully understood, and according to Rinklebe and Shaheen^60^, serious attention must be paid to this essential nutrient that, at high concentrations, can adversely affect organisms. Coprecipitation with Fe and Mn (hydr)oxides can be associated with Ni's potential to mobilize during changes in pH, increasing phase partitioning, and toxicity in water sediments^61^. Ni's high background content in the Carpathians was conditioned by river hydrological characteristics, and it is most often bound in immobile phases^62^.

Copper exceeded the prescribed limits at sites #2 (T1) and #12 (T3 and T4), indicating a low contamination risk. The interquartile ranges (q3‒q1) in T1 = 26.94 mg/kg, T2 = 30.13 mg/kg, T3 = 24.15 mg/kg, and T4 = 26.2 mg/kg demonstrated low midspread variability regardless of season. Copper deposits naturally in hot waters associated with volcanism, explaining the high concentrations in sites #4 and #5 in all sampling periods (T1‒T4). Waters in the Călimănești region (#4, #5) can be cold (< 20 °C), hypothermal (20‒34 °C), thermal (42‒43 °C), or geothermal (> 87 °C) and can contain sulfurous, chlorinated, sodium, calcium and magnesium. Copper in the Danube River (#12) can be associated with anthropogenic sources, given that the values there were the highest measured in this study. This trace element is characterized by a high degree of remobilization from water to sediments. Under extreme events (floods), it can precipitate with sulfides in an anoxic environment under extreme events (floods) that induce resuspension^63^.

In our study, cadmium, a highly toxic metal responsible for environmental and human severe diseases, was at the upper limit in sites #4 (T3, T4), #10 (T1, T3, T4), #14 (T1, T3, T4), and #20, #21 (T2, T3, T4). The mean values (mg/kg with standard deviation) were T1 = 0.42 ± 0.27, T2 = 0.49 ± 0.32, T3 = 0.53 ± 0.26 and T4 = 0.53 ± 0.28, with a minimum interquartile range (q3‒q1) in T1 (0.23) and maximum in T4 (0.55). Recent literature indicates a strong relationship between tourism and cadmium occurrence, which affects the whole globe, except Antarctica^64^, and this pattern can explain cadmium's presence in areas with intense tourism (Olănești, Căciulata, Ocnele Mari). Cadmium presence in the Olt River due to anthropogenic activities can be associated with industrial and municipal waste. Point sources such as industrial platforms (e.g., helicopter producer IAR Ghimbav) were frequently reported as sources of cadmium release into the water ecosystem of the Olt River basin^19^. Even so, Ni–Cd batteries, fossil fuel combustion, phosphate fertilizer, and waste incineration are responsible for Cd discharges in the environment^65^. The UNEP^66^ indicates that natural processes (e.g., dust storms, volcanic activities, climate change, erosion, and wildfire) are responsible for significantly higher Cd deposition than are anthropic processes. According to this study, Romania is included on a red list of high Cd contamination hotspots worldwide.

### Principal component analysis and hierarchical component analysis

**Figure 2:**
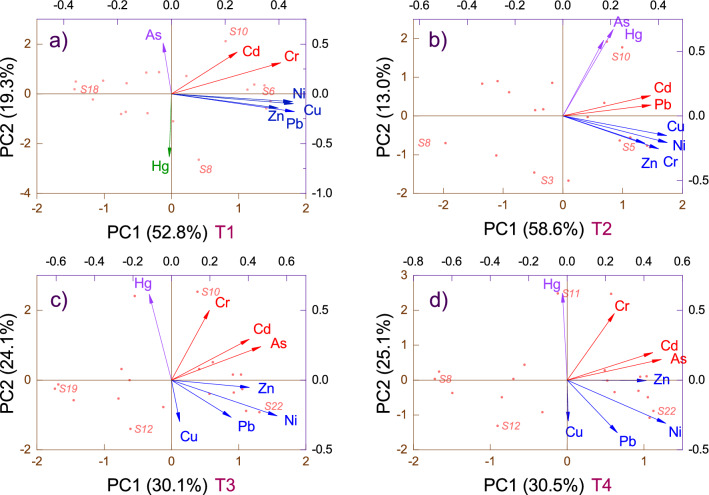
PCA of heavy metals in Olt River middle and lower riches measured during four periods of 2018 [**a**—March (T1); **b**—May (T2); **c**—June (T3); **d**—October (T4)].

River inputs and historical deposition upstream of dams are essential sources of toxic HM sediment contamination^19^. Remobilization between water and the bottom layers of sediments is regulated by water discharges of the Olt River and tributaries. PCA was applied to identify and interpret the metal relationships and discuss similar contamination sources^67^. The PCA results based on the correlation matrix for the four periods investigated are presented in Fig. 2. PCA output emphasized comparable amounts of variance explained by the first two vectors in March and May (PC1 = 52.8% and 58.6%; PC2 = 19.3% and 13.0%), respectively, and in June and October (PC1 = 30.1% and 30.5%; PC2 = 24.1% and 25.1%). The results showed that only the eigenvalues from the two components exceeded the threshold of 1 for March (PC1 = 4.22, PC2 = 1.54) and May (PC1 = 4.68, PC2 = 1.03). For June, four components were > 1 (PC1 = 2.40, PC2 = 1.92, PC3 = 1.33, PC4 = 1.05), and for October, there were three (PC1 = 2.44, PC2 = 2.00, PC3 = 1.38). In March, we observed different dominant origins for elements with loading grouped positively (As) and negatively (Hg) and possible common sources for Cd and Cr (moderately positive) and Cu, Zn, Ni, and Pb (moderately negative).

However, the loadings of the principal components in May were positive (As, Hg, Cd, Pb) and moderately positive (Cu, Ni, Cr, Zn). Comparable associations were observed for the loadings of principal components in June and October as positive (Cr, Cd, As), moderately positive (Cu, Pb, Ni, Zn), and moderately negative (Hg). Ni, Cu, Zn, Cr, and Pb are mainly derived from industrial effluents, domestic sewage, and runoff from mining and agriculture. However, they can be partially attributed to a natural geogenic origin from sediment accumulation and parental riverbeds. Cd, Hg, and As have loading factors in the first and second principal components, indicating that there is a mixed source that is less geogenic and more related to anthropogenic activities, a fact sustained even by correlation analysis and coefficients of variation. These metals are observed in different positions and with loadings of various lengths, suggesting one or more pollution sources. High loadings in PC2 were accumulated in this group associated with pesticides and phosphate fertilizers used in agricultural practices. Since toxicity and bioavailability are correlated with geochemical form and concentration, these elements can pose a potential hazard to the aquatic Olt River environment.

**Figure 3:**
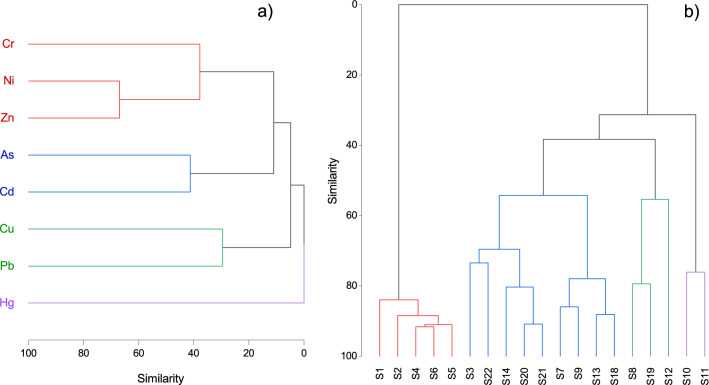
Dendrogram of hierarchical cluster analysis among heavy metals (**a**) and sites (**b**) (Euclidean distance, Wards method).

Divergent from most reported studies in which PCA and HCA were convergent here, HCA separated four groups according to the Ward cluster method and absolute correlation distance using similarity as the criterion (Fig. 3). Thus, the first group was associated Cr, Ni, and Zn, the second was associated As and Cd, the third was associated Cu and Pb, and the fourth was distinctly associated with Hg. These results suggested various levels of geogenic and anthropogenic origins of the related metals. The sampling location in the middle Olt River, an intramountain area that is mainly touristic due to its thermal waters, was related to one group with the highest mean site contamination level. Downstream, the Olchim Râmnicu Vâlcea chlor-alkali plant was included in a second group with many points indicative of anthropogenic activities associated with industries and municipal waste. The third group was associated the sampling location with a less distinct spatial pattern but with less contamination. Finally, the fourth group included two sites downstream from the Râmnicu Vâlcea municipality that were represented by artificial lakes with a similar pollution level.

### Correlation analysis

**Figure 4:**
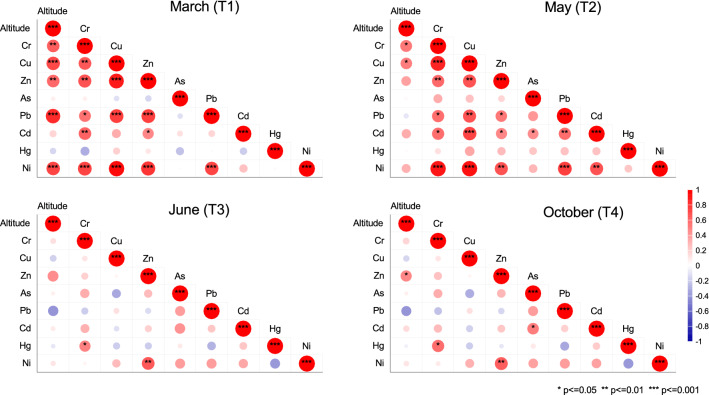
Pearson product-moment correlation inter-metals and between heavy metals and altitude from Olt River 2018 sediments.

Pearson correlation analysis indicated a strong significant positive relationship between site altitude and contaminant level only in March, with coefficient values of *r* = 0.82, *p* < 0.001 (Pb), *r* = 0.73, *p* < 0.001 (Ni), *r* = 0.70, *p* < 0.001 (Cu), *r* = 0.63, *p* < 0.01 (Cr) and *r* = 0.59, *p* < 0.01 (Zn). For the other periods analyzed, we observed low correlations in the cases of Cr (*r* = 0.53) and Cu (*r* = 0.51) in May and Zn (*r* = 0.48) in October (*p* < 0.05) (Fig. 4). It is worth noting that there was no significant relationship in June between any HM and elevation. Additionally, we observed that Pb was correlated negatively with elevation in both June (*r* = − 0.41, *p* = 0.08) and October (*r* = − 0.40, *p* = 0.08). Other research indicated a relationship between the decreasing trend of HM content and increases in elevation and river flow rate during the spring^68^. During the spring, the Pearson product-moment correlation coefficient demonstrated a strong (*p* < 0.001) intermetal relationship in March for Pb vs. Cu (*r* = 0.74), Pb vs. Zn (*r* = 0.75), Ni vs. Cr (*r* = 0.74), Ni vs. Cu (*r* = 0.95), and Ni vs. Zn (*r* = 0.80) and in May for Cd vs. Cu (*r* = 0.73), Ni vs. Cr (*r* = 0.90), and Ni vs. Cu (*r* = 0.92) (Fig. 4).

The correlations between elements indicated similar levels of HM contaminants, similar released sources of pollution, associated dependence during their transport, and remobilization in river sediments^69^. A high correlation coefficient between metals can indicate similar behavior under similar environmental conditions^70^. Moreover, the mean site concentration of Cr decreased from March (23 mg/kg) and May (43.57 mg/kg), which are rainy periods, to June (15.41 mg/kg) and October (17.84 mg/kg), which are mainly dry seasons. In this case, the lixiviation process can contribute to the dilution of HMs, as is often reported in the literature^71^. In contrast, the trend for Pb was the reverse, as it increased from March (22.46 mg/kg) and May (18.28 mg/kg) to June (58.35 mg/kg) and October (58.66 mg/kg). Gemeiner et al. and Eka et al. Also noted the relatively fast or slow Pb reaction quantified through the kinetic adsorption/desorption rate can explain the difference in elemental concentrations in the dry and wet seasons^72,73^.

### Pollution indices

Geoaccumulation index is used to assess the presence and intensity of anthropogenic contaminant accumulation in sediments. *I*_*geo*_ indicated a moderate level of Cu in June and October of 2018 (#12), when a reference geochemical crustal background was used. The *I*_*geo*_ calculated for Ni revealed sediment samples ranging from nonpolluted to moderately polluted, regardless of site and season. Except at locations #4, #9, #13, and #20, the As content was classified as moderate to heavy pollution in 2018 based on the Müller ranking^74^. Moderate contamination with Hg was observed at locations #10 and #11. The mean and standard deviation *I*_*geo*_ values presenting a negative result indicated no contamination for Zn, Cr, Cu, Ni, Pb, Cd, and Hg (− 2.0 ± 1.3; − 3.4 ± 2.0; − 2.0 ± 1.8; − 0.55 ± 0.9; − 2.1 ± 1.3; − 2.2 ± 1.7; and − 1.6 ± 1.2, respectively) and were positive for As (1.4 ± 1.4) in 2018. The *I*_geo_ for As indicated a change from moderate to strong pollution and 79% of samples ranged between 1.93 and 2.46. However, no significant differences between site locations with high-level metal contamination were detected. Pollution was observed for all sampling periods, suggesting that temperature increase rather than accidental discharge was the main factor associated with the increasing concentration.

*PI* index generally represents the toxicity status of elemental pollution in sediments and varies according to the crustal background used. Heavy pollution (*PI* > 3) was calculated for all sites except #3, #8, #12, and #19 in 2018. The mean *PI* values had ranges of 4.1 ± 1.6, 4.2 ± 1.8, 4.1 ± 1.7, and 4.1 ± 1.8 for March, May, June, and October, respectively. According to the *RI* values, an intermediate ecological risk was observed at locations #10 and #11 in May (216, 228), June (210, 195), and October (228, 206). Our study has illustrated more than inefficient use of participatory practices in the past regarding the spatiotemporal alteration of water bodies. It has also indicated possible negative influences via environmental drivers. Mining activities, agricultural practices, sewage discharge, and industrial discharge have exacerbated natural freshwater contamination, especially in west-central and southern Romania.

**Figure 5:**
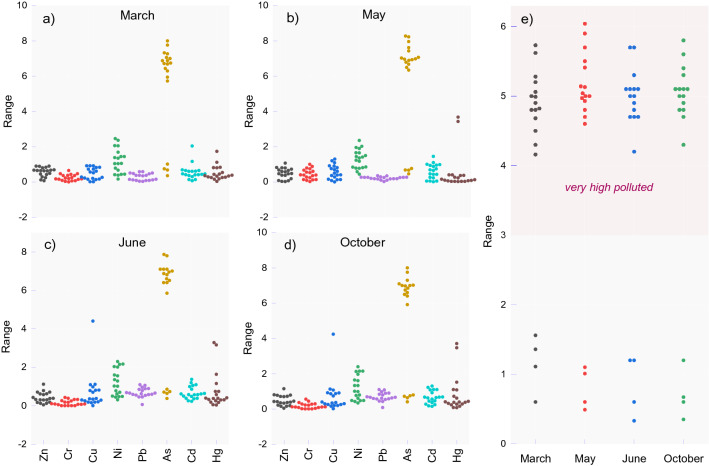
Contamination factor (CF) calculated for (**a**) March, (**b**) May, (**c**) June, (**d**) October, and Pollution Load Index (PLI) values (**e**) in the surface sediments of the Olt River graphical illustration.

*CF* index was assessed to estimate the degree of anthropogenic metal contamination levels of sediments in the Olt River during our four investigation periods in 2018. The *CF* values were established as the ratio of measured sample concentrations and the sediment background according to national regulations^36^. The *CF* values in the present survey varied in March as follows: 0.07‒0.90 (mean of 0.58) for Zn, 0.00‒0.65 (mean of 0.54) for Cr, 0.01‒0.91 (mean of 0.47) for Cu, 0.16‒2.42 (mean of 1.19) for Ni, 0.03‒0.58 (mean of 0.28) for Pb, 0.07‒2.04 (mean of 0.55) for Cd, and 0.03‒1.73 (mean of 0.49) for Hg. Thus, we interpreted the results as showing a low degree of contamination for Zn, Cr, Cu, and Pb. For Ni (#2 to #12, #20), Cd (#10, #14), and Hg (#8, #9), we judged there to be moderate levels of contamination (Fig. 5).

Extreme values quoted as "very high" (i.e., until *CF* = 8.00) were assessed for As at 13 sites (#1, #4, #5, #6, #7, #10, #11, #13, #14, #18, #20, #21, #22) of the 19 sites investigated. At sites #2 and #9, "considerable contamination" was found, and at sites #3, #8, #12 and #19, the *CF* did not exceed the moderate level (*CF* = 1.01). Contamination factors in March had ranges of 0.02‒1.07 (mean of 0.49) for Zn, 0.02‒1.00 (mean of 0.44) for Cr, 0.00‒1.30 (mean of 0.56) for Cu, 0.44‒2.35 (mean of 1.30) for Ni, 0.02‒0.35 (mean of 0.22) for Pb, 0.01‒1.45 (mean of 0.60) for Cd, and 0.02‒3.86 (mean of 0.52) for Hg. Moderate contamination was found for Zn (#22), Cr (#5), Cu (#2, #4, #5), Ni (#2, #3, #4, #5, #6, #9, #10, #11, #12, #14, #20, #21) and Cd (#4, #20, #21).

A considerable degree of pollution was calculated for Hg (#10, #11), and high As contamination was detected at all sites (range of 6.34‒8.28), except for sites #3, #8, #12 and #19 (range of 0.46‒0.76). In June, the average *CF* values were comparable to those from March and May, as follows: 0.44 (Zn), 0.15 (Cr), 0.71 (Cu), 1.20 (Ni), 0.69 (Pb), 5.25 (As), 0.67 (Cd), and 0.77 (Hg). Moderate contamination was detected for Zn (#22), Cu (#4, #5), Ni (#1, #2, #4, #5, #6, #12, #13, #14, #20, #21, #22), Pb (#13, #14), Cd (#4, #10, #14, #20, #21), and Hg (#8, #9). Considerable contamination levels were measured for Cu (#12) and Hg (#10, #11). Except for sites #3, #8, #9, and #12, all the sampling sites were highly polluted with As. In October, the data revealed results similar to June’s, in which values were greater than 3, which indicated considerable and high contamination degrees. The *RI* index indicated middle ecological risk in May, June and October only for sites #10 and #11.

The *PLI* was investigated to compare the integrated pollution level at different sampling sites, as HMs can significantly vary in various sediment samples. The *PLI* was assessed as the *n*^*th*^ root of the multiplication of contamination factor, where values > 1 qualified the site as polluted^75^. We found the *PLI* values to have ranges of 0.06‒5.73 (cv = 40%) in March, 0.49‒6.04 (*cv* = 44%) in May, 0.33‒5.7 (*cv* = 43%) in June, and 0.35‒5.8 (*cv* = 45%) in October, indicating that over 89% of the sites were polluted by HMs to various degrees (Fig. 5). The most polluted site was #11 (Drăgășani accumulation lake), located downstream from the Râmnicu Vâlcea municipality. Iordache et al. conducted a study near the Râmnicu Vâlcea upstream chlor-alkali plant and reported a low level of *CF* for most sites with Ni, Cu, Pb, and Cu^76^; the authors also calculated the *PLI,* which indicated no HM enrichment in surface sediments. However, surveys conducted in 2015 located between Călimănești (#4) and downstream Râmnicu Vâlcea, near our site #22, indicated moderate and considerable contamination of Cu, Hg, Zn, Ni, and Cr and serious levels of pollution at 7 of 9 sites^26^. Furthermore, in 2019, the investigated sites were considerably or highly contaminated with As, according to the *CF*. According to the *PLI* eight locations from 22 that were analyzed were relatively highly polluted^25^. These results show enrichment in the last ten years of Olt River surface sediments. Thus, according to reviewed reports, there has been moderate pollution found downstream from the chlor-alkali plant over the past ten years. At present, we found high surface sediment contamination in both the middle and the lower reaches of the Olt River.

## Conclusions

HM concentrations in southern Romania and the Olt River surface sediment samples were investigated to assess their spatial distribution, seasonality, and contamination levels. The results showed that the concentrations of Ni, Cu, Zn, Pb, Cd, and Hg in surface sediments were generally higher than their respective national background values—up to 8.28 times more in the case of As. The spatial variability pattern had similar trends for all HMs, indicating increasing concentrations in the middle compared to the lower sites. The statistical tests showed a statistically significant difference between the mean and variance only for Cr and Pb when investigating the temporal variability between site measurements. We observed a strong association between HMs in March and May and a strong relationship in March between elevation and Pb, Ni, Cu, Cr, and Zn, demonstrating that river velocity accumulated with spring discharges are conditioning metal levels in the spring. The HMs Cd, Hg, and As had mixed sources that were less geogenic and more from anthropogenic activities. The *CF* indicated that the Olt River was highly contaminated with As at 15 of 19 sites. A low degree of contamination was also observed in various locations for the investigated elements. *PI* analysis indicated that the Olt River sediments were highly polluted (*PI* > 3), and the *PLI* showed varying degrees of pollution in more than 89% of the sites surveyed, increasing the authorities' need for action. The *I*_*geo*_ indicated that Zn, Cr, Cu, Ni, Pb, Cd, and Hg were at a pollution-free level and that As was present at levels ranging from unpolluted to moderately polluted. A higher involvement by national authorities, including monitoring measures to control contamination in industrial areas and municipal waste discharges, is needed to improve the Olt River environment.

## Supplementary Information

Supplementary Information.
