# Supplementary material for: Accumulation and ecotoxicological risk assessment of heavy metals in surface sediments of the Olt River, Romania
Source: Sci Rep. 2022 Jan 18;12:880. doi: 10.1038/s41598-022-04865-0 (PMC8766583; doi:10.1038/s41598-022-04865-0)
Supplement: Supplementary file 1 — Supplementary Information. [file 41598_2022_4865_MOESM1_ESM.docx]

**Supplementary Information**

**Supplementary Methods**

**2.1. Analytical procedure**

For sample consolidated sediment consisting of silt and sand, a Van Veen grab with a capacity of 12 liters was used. The decomposition of solid materials by acids in closed vessels at elevated temperatures and pressures assisted by microwave radiation is used as a sample preparation technique in the environmental field. The sediments were transported in high-density polyethylene bottles of one-liter size, in accordance with standard sampling guidelines (SR EN ISO 5667-13, 2011). This essential step aims to change the physical form of the geological material, to make it compatible with the chosen analytical technique without changing its chemical composition. The bottles were rinsed with deionized water (DI) and soaked in 5% nitric overnight for storing the samples. The sediment samples were further dried at 65°C for 12 h, homogenized, and sieved with 2 mm mesh. Finally, 0.5 g of sediment was digested using a closed iPrep vessel speed iWave system MARS 6 CEM One Touch (CEM Corporation, Matthews, North Carolina, USA) with a mixture of concentrated acids (9 ml nitric acid 69% + 1ml HF 48%). Two steps temperature-controlled digestion program microwave digestion of sediments (Buffalo River, Leach from CEM Mars 6 Method Note Compendium, 2018).

The greater sensitivity and broader elemental coverage offered by inductively coupled plasma mass spectrometry (ICP-MS) made it, from its earliest days, an attractive and potentially powerful technique for determining trace elements in geological materials. The determination of the elements Zn, Cr, Cu, Ni, Pb, As, Cd, and Hg in sediment were performed using an inductively coupled plasma mass spectrometer, ICP-QMS 820-MS (Varian, Melbourne, Australia), equipped with an SPS-3 autosampler (Varian, Malgrave, Australia) and collision-reaction interface, iCRI, working in H_2_ and He modes. The optimal conditions were as follows: RF power of 1400 W, plasma gas-flow rate of 12 L/min, auxiliary gas-flow rate of 1.5 L/min, nebulizer gas-flow rate of 1.05 L/min, H_2_ gas flow rate of 90 mL/min, He gas flow of 90 mL/min, and a dwell time of 2 ms for ^52^Cr, ^60^Ni, ^66^Zn, ^75^As, ^111^Cd, ^202^Hg, ^208^Pb, and ^209^Bi, with ^6^Li, ^45^Sc, ^159^Tb, and ^89^Y isotopes serving as internal standards. Certipur® ICP multi-element standard solution XXI, Certipur® ICP multi-element standard solution IV, concentrated HNO_3_ 69% (w/v), concentrated HF (48%) and HCl (37%), reagent grade (Merck, Darmstadt, Germany), and ultrapure water with a maximum resistivity of 18.2 MΩ cm, were used for the preparation of calibration curves (a maximum of hundredfold dilution was used). All the investigated calibration curves were characterized by a high correlation coefficient (*r*>0.995) for both analytical methods.

*2.2. Assessment of sediment pollution level*

*2.2.1. Geoaccumulation index* (*I_geo_*) was defined initially by Müller (1979) as equation 1, where $C_{n}$ represents the measured content of an element in sediments (mg/kg); $B_{n}$ represent the geochemical background value (mg/kg), and the constant (1.5); allows to analyze natural environmental fluctuation for a specific trace element and detect trace anthropogenic influence. The *I_geo_* indices are logarithmic, being hard to identify the minor contamination and reliable for quantifying sediments with significant enrichment from urbanization and industrial activities.

$I_{geo}= {log}_{2} \left( \frac{C_{n}}{1.5B_{n}} \right)$…………………. (Eq. 1)

The interpretation of I_geo_ index values are categorized into seven levels (0-6, Müller, 1981)

*2.2.2. Nemerow Pollution Index* (PI) also indicates the quality of sediments (Nemerow, 1991) by assessing the most critically potentially toxic elements. The PI can overcome the issues identified in the modified degree of contamination index by estimating trace elements' single and combined pollution levels. The weighted average equation that considers the average contamination factors of a suite of features (PI_avg_) and the Impact of maximum contamination factor (PI_max_) allows evaluating a single component's effect.

$PI =\sqrt{\frac{P_{i avg}^{2}+ P_{i max}^{2}}{2}}$ …………………. (Eq. 2)

*P_i_* = C_i_ / S_i_ …………………. (Eq. 3)

*C_i_* represents the measured content of the target element (mg/kg), and *S_i_* is the investigated element's background value (mg/kg). Therefore, the Nemerow index was interpreted as the following: PI ≤0.7 – grade security; 0.7<PI≤0.1 – minimal impact; 1<PI≤0.1 – small impact; 2<PI≤0.1 – moderate impact; PI>3 – heavy Impact.

*2.2.3. Potential Ecological Risk* (RI) was reported by Hakanson (1980) as an ecological risk index for aquatic pollution control. The method is used in environmental risk assessment as a diagnostic tool to penetrate one of many possible avenues towards a potential hazardous ecological risk index.

$C_{f}^{i}= C^{i}/C_{n}^{i}$ …………………. (Eq. 4)

$E_{r}^{i}= T_{r}^{i}* C_{f}^{i}$ …………………. (Eq. 5)

$RI=\sum_{i=1}^{n} E_{r}^{i}$ …………………. (Eq. 6)

where $C_{f}^{i}$ is the metal contamination index, $C^{i}$ – represents the average content for metal determination (mg/kg), $C_{n}^{i}$ – is the local background level value of heavy metals (mg/kg) before the industrialization period. In our case the elemental background values, due to lack of data, will be assimilated with national regulation, for Zn (150 mg/kg), Cr (100 mg/kg), Cu (40 mg/kg), Ni (35 mg/kg), Pb (85 mg/kg), As (29 mg/kg), Cd (0.8 mg/kg), and Hg (0.3 mg/kg). $E_{r}^{i}$ indicate the potential ecological risk factor and $T_{r}^{i}$ – represents the toxicity coefficient of a single metal, which were defined as 30 (Cd), 2 (Cr), 5 (Cu, Pb), 6 (Ni), 1 (Zn), 40 (Hg), and 10 (As).

Hakanson established four levels of pollution: $C_{f}$< 1 (low contamination); 1 ≤ $C_{f}$< 3 (moderate contamination); 3 ≤ $C_{f}$< 6 (considerable contamination);$C_{f}$ ≥6 (very high contamination). The *CF* reveals information for each element, and the sum of the contamination factors for all features represents the contamination degree ($C_{deg}$) of the site investigated. $C_{deg}$ < 8 represents a low degree of contamination; 8 ≤ $C_{deg}$< 16 indicates a moderate contamination; 16 ≤ $C_{deg}$< 32 means that the level of contamination is considerable; $C_{deg}$≥$32$signifies a very high degree of contamination.

*2.2.4. The pollution load index* (PLI) indicates the toxicity of all heavy metals in one site Eq. (7). According to Tomlinson et al. [25], the *PLI* values were subsequently calculated from the contamination factors to minimize the possible anthropogenic influences.

*PLI* = ${(CF1 \times CF2 \times CF3 \times\ldots CFn)}^{1/n}$ …………………. (Eq. 7)

*PLI* > 1 indicates pollution and *PLI* < 1 implies unpolluted surface sediments.

**Supplementary Results and Discussion**

Supplementary Table 1. Geographical localization and summary statistics for heavy metals and trace elements in 2018 sediments were presented as the median values.

| Site | Latitude (N) | Longitude (E) | Zn (mg/kg) | Cr (mg/kg) | Cu (mg/kg) | Ni (mg/kg) | Pb (mg/kg) | As (mg/kg) | Cd (mg/kg) | Hg (mg/kg) |
| --- | --- | --- | --- | --- | --- | --- | --- | --- | --- | --- |
| #1 | 45°23'32" | 24°17'42" | 105.35 | 44.60 | 36.80 | 71.30 | 49.10 | 191.22 | 0.49 | 0.08 |
| #2 | 45°20'50" | 24°16'34" | 113.52 | 36.31 | 32.91 | 59.42 | 55.80 | 188.90 | 0.74 | 0.11 |
| #3 | 45°16'47" | 24°18'42" | 59.73 | 37.54 | 30.16 | 49.35 | 55.90 | 19.58 | 0.52 | 0.07 |
| #4 | 45°14'35" | 24°20'55" | 123.27 | 42.63 | 44.40 | 70.63 | 53.40 | 198.66 | 0.84 | 0.11 |
| #5 | 45°11'08" | 24°21'33" | 117.54 | 46.50 | 44.10 | 82.39 | 58.40 | 224.80 | 0.54 | 0.12 |
| #6 | 45°07'13" | 24°22'18" | 103.50 | 46.59 | 35.94 | 68.34 | 51.50 | 203.40 | 0.54 | 0.22 |
| #7 | 45°00'33" | 24°18'30" | 52.30 | 11.40 | 15.20 | 20.29 | 37.90 | 203.60 | 0.37 | 0.02 |
| #8 | 45°55'17" | 24°14'38" | 49.75 | 1.76 | 13.80 | 16.70 | 45.80 | 24.80 | 0.21 | 0.49 |
| #9 | 44°51'35" | 24°14'47" | 65.04 | 14.88 | 20.26 | 36.96 | 49.20 | 171.86 | 0.53 | 0.33 |
| #10 | 44°46'46" | 24°16'32" | 91.55 | 36.80 | 22.41 | 48.63 | 36.80 | 199.40 | 1.10 | 1.03 |
| #11 | 44°41'13" | 24°17'32" | 29.93 | 43.00 | 8.10 | 27.05 | 35.25 | 231.40 | 0.52 | 1.04 |
| #12 | 43°42'33" | 24°46'24" | 52.90 | 22.40 | 70.53 | 46.80 | 67.40 | 21.02 | 0.31 | 0.17 |
| #13 | 43°44'10" | 24°47'50" | 30.15 | 13.09 | 10.40 | 34.70 | 73.56 | 214.70 | 0.31 | 0.12 |
| #14 | 43°43'23" | 24°53'34" | 56.84 | 32.60 | 12.14 | 35.70 | 90.20 | 201.50 | 0.89 | 0.13 |
| #18 | 44°10'10" | 24°28'12" | 9.72 | 1.00 | 6.22 | 21.20 | 16.87 | 192.70 | 0.30 | 0.09 |
| #19 | 44°16'09" | 24°24'04" | 64.67 | 12.30 | 11.24 | 27.41 | 7.20 | 11.95 | 0.20 | 0.11 |
| #20 | 44°23'52" | 24°20'53" | 68.90 | 11.20 | 31.50 | 75.40 | 75.50 | 185.50 | 1.02 | 0.22 |
| #21 | 44°27'15" | 24°19'16" | 51.51 | 4.00 | 32.65 | 44.11 | 44.11 | 202.80 | 0.85 | 0.06 |
| #22 | 44°32'28" | 24°20'03" | 164.33 | 10.14 | 7.70 | 27.02 | 14.98 | 202.70 | 0.37 | 0.02 |

Supplementary Table 2. Comparison of heavy metal concentration of river sediments in the Olt River with other areas around the world. (unit: mg/kg).

| Location | Cr | Ni | Cu | Zn | As | Pb | Cd | Hg | Reference |
| --- | --- | --- | --- | --- | --- | --- | --- | --- | --- |
| Study area | 100.66 | 86.31 | 176 | 172.9 | 240.14 | 94.2 | 1.23 | 1.11 | This study |
| Pra Basin of Gana | 216.70 | 79.92 | ‒ | 35.62 | 0.15 | 335.38 | 3.20 | ‒ | Duncan et al., 2018 |
| Pra Basin of Gana | 3.614 | 1.177 | 1.155 | 5.906 | 0.714 | 2.441 | ‒ | 2.917 | Donkor et al., 2005 |
| Lijiang River, China | 43.62 | 22.95 | 31.72 | 129.33 | 18.3 | 42.8 | 0.97 | 0.39 | Xiao et al., 2021 |
| Lake Taihu China | 102.46 | 45.50 | 44.71 | 163.62 | 13.34 | 37.00 | 0.479 | 0.109 | Zhang et al., 2019 |
| Lake Taihu China | 147.5 | 79.5 | 97.5 | 223.1 | 21.4 | 133.2 | 1.97 | 0.34 | Niu et al., 2020 |
| Seine River, France | 123 | 31 | 99 | 370 | ‒ | 108 | 1.8 | 1.08 | Meybeck et al., 2007 |
| Seine River, France | 55 | 21 | 68 | 152 | 7.3 | 83 | 0.6 | ‒ | Le Gall et al., 2018 |
| Odra River Germany / Poland | 47.5 | 19.1 | 88.3 | 471 | ‒ | 163 | 7.87 | 2.99 | Boszke et al., 2004 |
| Danube River Romania | 99.87 | 99.67 | 126.52 | 217.43 | 16.96 | 84.75 | 1.33 | 0.52 | Radu et al., 2019 |
| Hackensack River United States | 207.52 | 50.94 | 136.17 | 328.59 | ‒ | 160.72 | 6.61 | 4.29 | Artigas et al., 2017 |
| East Riser  United States | 93.96 | 36.61 | 113.38 | 414.66 | ‒ | 156.62 | 6.06 | 8.95 | Artigas et al., 2017 |
| Peach Island East Unites States | 426.90 | 136.20 | 433.94 | 885.08 | ‒ | 207.73 | 24.59 | 51.78 | Artigas et al., 2017 |
| Kuril-Kamchatka  Russia | 48.3 | 59.9 | 61.1 | 119.9 | 13.14 | 25.1 | 0.6 | ‒ | Sattarova and Aksentov, 2021 |
| Lower Indus Pakistan | 109 | 71.59 | 34.44 | 150 | 14.45 | 45.88 | 6.95 | ‒ | Nawab et al., 2018 |
